# Cilostazol mitigates amiodarone-induced pulmonary toxicity and fibrosis by regulating the cAMP/TGF-β1 pathway-mediated epithelial-to-mesenchymal transition in rats

**DOI:** 10.1038/s41598-026-45341-3

**Published:** 2026-05-13

**Authors:** Mohamad A. El-Gammal, Eman H. Yousef, Ahmed G. Abd Elhameed, Mohamed M. Salama, Muhammed M. Salahuddin

**Affiliations:** 1 Pharmacology and Biochemistry Department (Pharmacology), Faculty of Pharmacy, Horus University-Egypt, New Damietta, 34518 Egypt; 2 Pharmacology and Biochemistry Department (Biochemistry), Faculty of Pharmacy, Horus University-Egypt, New Damietta, 34518 Egypt; 3 Pharmacology and Toxicology Department, Faculty of Pharmacy, Mansoura University, Mansoura, 35516 Egypt; 4 Biochemistry Department, Faculty of Pharmacy, Delta University for Science and Technology, Gamasa, 11152 Egypt

**Keywords:** Amiodarone, Fibrosis, Cilostazol, Transforming growth factor beta-1 (TGF-β1), SIRT1, Epithelial-to-mesenchymal transition (EMT), Cell biology, Diseases, Drug discovery, Medical research, Molecular biology, Molecular medicine

## Abstract

Cilostazol, a selective phosphodiesterase type III inhibitor, enhances intracellular cAMP and exhibits potent antioxidant and anti-inflammatory properties. This study aimed to investigate the protective effect of cilostazol (50 mg/kg/day, orally) against amiodarone (AMIO)-induced pulmonary fibrosis (PF) and to elucidate its underlying mechanisms. Lung index as well as total and differential cell counts in bronchoalveolar lavage fluid (BALF) were estimated. Histopathological changes were evaluated using hematoxylin and eosin (H&E) and Masson’s trichrome staining. Malondialdehyde (MDA) and glutathione (GSH) contents were assessed colorimetrically, while TNF-α, IL-1β, and cAMP levels in the lungs were determined using ELISA. TGF-β1 and vimentin expressions were examined immunohistochemically, SIRT1 protein expression by Western blotting, and EPAC1 gene expression by RT-PCR. AMIO administration caused significant increases in lung index, inflammatory cytokines, oxidative stress markers, and fibrotic mediators, accompanied by a decline in GSH and cAMP levels and severe histopathological damage. Cilostazol co-treatment markedly attenuated these alterations, decreasing TNF-α, IL-1β, MDA, TGF-β1, and vimentin while restoring GSH, cAMP, SIRT1, and EPAC1 expressions and improving lung architecture. Cilostazol mitigates AMIO-induced PF through attenuation of oxidative stress, inflammation, and EMT, potentially via activation of the SIRT1/EPAC1/cAMP pathway. These findings highlight cilostazol as a promising therapeutic adjunct against drug-induced PF.

**Supplementary Information:**

The online version contains supplementary material available at 10.1038/s41598-026-45341-3.

## Introduction

Interstitial lung diseases (ILDs) are a distinct group of disorders affecting lung parenchyma, characterized by varying degrees of inflammation and fibrosis. Among them, idiopathic pulmonary fibrosis (IPF) is the most extensively studied. A hallmark of IPF is lung tissue scarring, in which fibroblasts and myofibroblasts replace alveolar epithelial cells^1–3^. The pathophysiology of pulmonary fibrosis (PF) is complex, involving environmental factors, autoimmune conditions, and genetic anomalies^2,4^, with a key pathological feature being a dysregulated wound-healing response following epithelial injury^5^. Clinically, PF manifests as breathlessness, coughing, hypoxia, fatigue, and can ultimately lead to respiratory failure, lung cancer, and death^2,4^. PF progression diminishes lung function and disrupts pulmonary architecture, resulting in poor clinical outcomes with a median survival of 2–3 years^6,7^. The prevalence and incidence of PF increase with age, with most cases diagnosed in patients over 60^8^. IPF, the most common form of PF, has shown a rising prevalence from 42.7 to 63 cases per 100,000 people, with an annual incidence of 16.3–17.4 cases per 100,000 people^9,10^. IPF primarily affects older adults, with a mean age of onset of approximately 65 years and a survival rate of 3–5 years after diagnosis^11,12^. While immunosuppressive therapies and corticosteroids have demonstrated some effectiveness, they are associated with severe side effects and only marginal improvements in survival^6,13^.

Amiodarone (AMIO), a powerful antiarrhythmic medication, is linked to lung damage that can lead to lethal PF^14^. Although the mechanism of AMIO-induced lung fibrosis is well understood, managing this lung disease is often challenging, and there are unfortunately few pharmacological strategies to prevent the development of AMIO-induced lung fibrosis^15^.

A key mechanism driving fibrosis in IPF is the epithelial–mesenchymal transition (EMT), an evolutionarily conserved process in which epithelial cells acquire a mesenchymal phenotype^16,17^. EMT is characterized by the loss of epithelial adhesion molecules, such as E-cadherin, and/or the gain of mesenchymal markers, including N-cadherin, vimentin, and alpha-smooth muscle actin (αSMA)^18,19^. This Janus-faced process plays a pivotal role in normal embryogenesis and wound healing, while also contributing to chronic pathologies such as fibrosis and cancer^20^. Although EMT has been extensively studied in lung^21^ and cancer^22,23^, but it has also been linked to chronic human lung and airway diseases such as chronic obstructive pulmonary disease (COPD) and IPF^24–27^. Alveolar epithelial cells (AECs) undergoing EMT^28,29^ or partial EMT^30^, can serve as a source of extracellular matrix-producing fibroblasts and myofibroblasts, driving tissue remodeling and fibrosis.

Among the signaling pathways regulating EMT and fibroblast function, cyclic adenosine monophosphate (cAMP) has emerged as a key modulator^31–33^. The second messenger cAMP is as a regulator of fibroblast function. cAMP is generated by adenylyl cyclases (ACs) in response to G-protein-coupled receptor (GPCR) activation and is degraded by cyclic nucleotide phosphodiesterases (PDEs)^34^. It exerts its effects primarily through protein kinase A (PKA), exchange protein directly activated by cAMP (Epac), and cyclic nucleotide-gated (CNG) ion channels^34^. 

 The cAMP effector, Epac ^35–37^, has been identified as an important mediator of the antifibrotic effect of cAMP. The two forms of Epac, Epac1 and Epac2, respectively possess one and two cAMP binding sites and function as guanine nucleotide exchange factors for the low molecular weight G-protein Rap (e.g. Rap1 and Rap2). Epac proteins regulate numerous cellular responses through their ability to promote the exchange of GTP for GDP on Raps and perhaps other G-proteins^35–37^. cAMP analogues that selectively activate PKA or Epac^38,39^ can aid in defining their role as effectors of cellular responses.

Cilostazol, a selective phosphodiesterase type III (PDE III) inhibitor, elevates intracellular cyclic adenosine monophosphate (3′,5′-cAMP) levels^40^. It is an FDA-approved vasodilatory antiplatelet agent clinically used for the management of intermittent claudication in the United States^40,41^ and for reducing the recurrence of cerebral thrombosis and lacunar stroke in Japan^42^. In addition to increasing cAMP, cilostazol has also been reported to elevate cyclic guanosine monophosphate (cGMP) levels^43^.Beyond its vasodilatory and antiplatelet activities, cilostazol exerts anti-inflammatory effects by inhibiting cytokine-induced expression of various pro-inflammatory and adhesion molecule genes^44^. Furthermore, cilostazol prevents oxidative stress by activating cellular redox defense systems through upregulation of phosphoinositide-3 kinase/protein kinase B (PI3K/Akt) and nuclear factor erythroid-2-related factor/heme oxygenase-1 (Nrf2/HO-1) mRNAs, thereby reducing oxidative stress and restoring mitochondrial dysfunction^45^.

The beneficial pharmacological effects of cilostazol are largely attributed its antioxidant^46^, anti-inflammatory and antiapoptotic properties^47^. Consistent with these actions, cilostazol has demonstrated protective effects in several experimental models of tissue injury, including toxic liver injury induced by CCl4^48^ and ischemia/reperfusion^49^. Additionally, cilostazol treatment has been shown to decrease the cellularity of the lung interstitium, pulmonary edema, and malondialdehyde (MDA) levels in lung tissue following limb ischemia/reperfusion^50^. However, mounting data from research has shown that elevated intracellular cAMP plays a crucial function in preventing fibrosis^51,52^, it is yet unclear how cilostazol prevents lung fibrosis. The current study sought to determine if cilostazol could protect rats against AMIO-induced lung damage and fibrosis, with a focus on the function of the cAMP/TGF-β1–mediated epithelial-to-mesenchymal transition pathway.

##  Materials and methods

### Drugs and chemicals

AMIO (Cordarone) was obtained from Global Napi Pharmaceutical Co., Egypt (manufactured under license from Sanofi Aventis, France). AMIO was administered using tablets containing 200 mg of amiodarone hydrochloride. Cilostazol was provided as a commercial preparation (Pletaal^®^; 100 mg tablets, Otsuka Pharmaceutical Co., Ltd., Tokyo, Japan). All additional chemicals and dietary components utilized in the study were of high purity and analytical grade.

### Animals

Eighteen male Sprague–Dawley rats (200 ± 20 g) were procured from the Egyptian Organization for Biological Products and Vaccines (Giza, Egypt). The animals were allowed a one-week acclimatization period under standard laboratory conditions, with unrestricted access to food and water, a 12-h light/dark cycle, and a controlled ambient temperature of 25 ± 1 °C. All experimental procedures were reviewed and approved by the Research Ethics Committee of Horus University–Egypt (Ethical code: PH-2025-005) and were conducted in accordance with the “Guide for the Care and Use of Laboratory Animals” (National Research Council, 8th edition, USA, 2011). This study was carried out in accordance with the ARRIVE guidelines.

###  Induction of pulmonary fibrosis

PF was induced by oral administration of 50 mg/kg AMIO^53^ daily for 5 weeks. A preliminary study was conducted, and relevant literature was reviewed to determine the optimal dose and duration for the induction of lung fibrosis. According to previous reports, AMIO doses ranging from 30 to 60 mg/kg/day administered for 3 to 10 weeks have been used to establish experimental lung fibrosis^53–56^. Based on these findings, a pilot study was performed using a dose of 50 mg/kg/day for 4 weeks, 5 weeks, and 5 weeks followed by a one-week period prior to sacrifice. The latter regimen (50 mg/kg/day for 5 weeks with an additional week before sacrifice) proved to be the most effective for establishing a reproducible lung fibrosis model.

### Experimental design

**Fig. 1:**
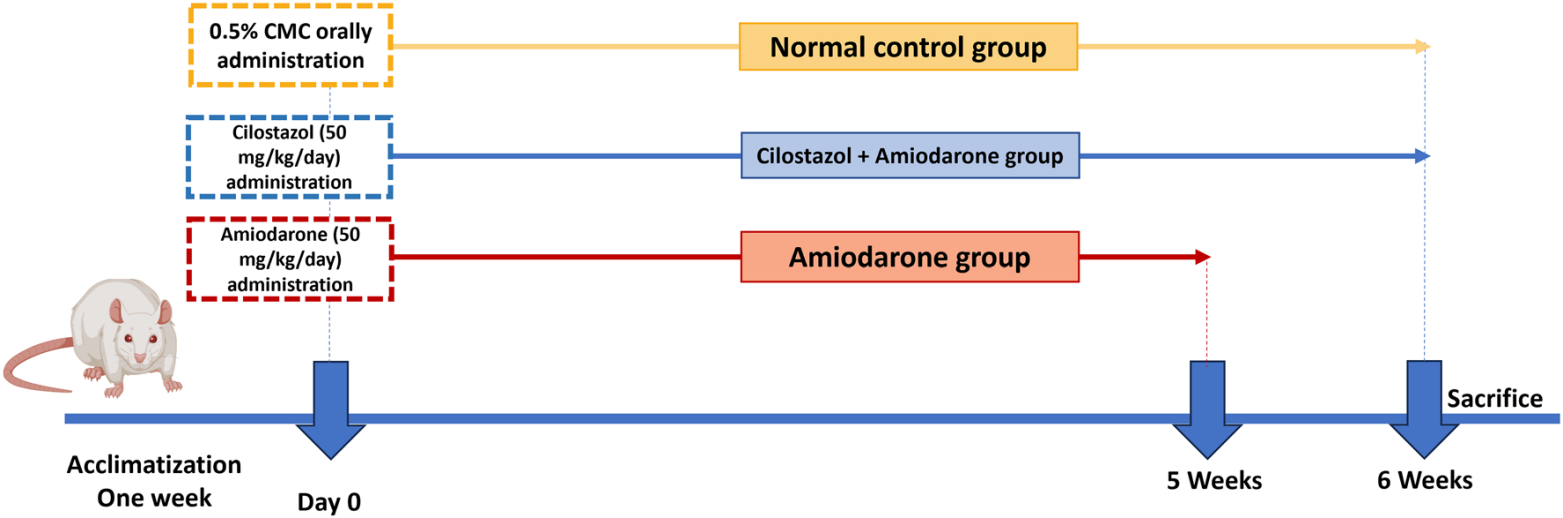
Schematic overview of the in vivo experimental research protocol.

The experiment was conducted over a six-week period. A schematic representation of the study timeline and design is provided in Fig. 1. Following a one-week acclimation phase, the 18 rats were randomly divided into three groups, each consisting of six animals:

Group (1): The normal (control) group, which was given 0.5% CMC orally.

Group (2): AMIO-induced group (fibrosis model): Rats were administered amiodarone orally at a dose of 50 mg/kg per day for five weeks^55,57^.

Group (3): Cilostazol-treated group: Rats received cilostazol orally at a daily dose of 50 mg/kg for six weeks, administered one hour prior to each AMIO dose^58^.

###  Blood and tissue preparation

At the end of the experiment (24 h after the last dose of cilostazol), the rats were weighed, kept fasting overnight, and then deeply anesthetized with pentobarbital sodium (40 mg/kg, i.p.)^59^. Once deep anesthesia was confirmed by loss of reflexes, bronchoalveolar lavage fluid (BALF) was collected under anesthesia. During this procedure, the left lung was ligated at the hilus to prevent dilution of lavage fluid, and BALF was obtained from the right lung via tracheal cannulation. Following BALF collection from right lung, animals were euthanized by exsanguination via thoracoabdominal incision performed by trained personnel. The left lung of each animal was then excised and sectioned into three parts. One part was fixed in 10% neutral-buffered formalin for histopathological and immunohistochemical examinations. Another part was quickly snap-frozen in liquid nitrogen for qRT-PCR analysis. The remaining portion was homogenized in four volumes of phosphate-buffered saline (PBS, pH 7.4) to prepare a 10% tissue homogenate, which was centrifuged at 8000 rpm for 20 min at 4 °C. The obtained supernatant was stored at − 80 °C for later biochemical analyses.

### Estimation of lung index

Lung tissues were meticulously excised and cleared of all adherent fat. The relative lung weight was subsequently determined according to the following equation:

$${\text{Relative lung weight }} = \left( {{\text{lung weight in grams/body weight}}} \right) \times 100.$$

### Collection of bronchoalveolar lavage fluid (BALF)

BALFwas collected as previously described^60^. Briefly, the left lung of each rat was ligated at the hilus, and an 18-gauge catheter was inserted via the trachea into the right main bronchus. BALF was collected from right lung by instilling and retrieving 2 mL aliquots of sterile saline. The recovered fluid (yielding 50–70%) was centrifuged at 2000 rpm for 10 min at 4 °C. Supernatants were discarded, and the resulting cell pellets were resuspended in PBS. Total cell counts were subsequently determined using a hemocytometer^61^.

###  Histopathological examination of lung tissue

For histological, histochemical, and immunohistochemical assessment, lung tissues were fixed in 10% neutral-buffered formalin, dehydrated through graded ethanol, and embedded in paraffin. Five-micrometer sections were stained with hematoxylin and eosin (H&E) and Masson’s trichrome. Images were acquired using a digital camera–equipped system (Nikon, Japan) and examined with an Olympus BX51 optical microscope (Olympus Corporation, Tokyo, Japan). Scoring system was done with modification according to Passmore et al.^62^ and shown in Table 1. The average area percentage of blue-stained collagen bundles in each group was calculated using Masson’s trichrome stained sections.

**Table 1:** Lung histology scoring system.

Score	Necrotic changes	Fibrosis	Inflammation
0	None	None	None
1	Minimal; few bronchiolar necrosis	Minimal	Few, rare inflammatory cells
2	Mild to moderate bronchiolar necrosis	Mild to moderate	Mild, focal peribronchiolar or interalveolar inflammation
3	Diffuse; many necrotic areas	Diffuse, severe	Moderate to severe; coalescing inflammatory aggregates

### Immunohistochemical analyses

For immunohistochemical analysis, antibodies against TGF-β1 (Cat. No. ab215715; Abcam) and Vimentin (Cat. No. A2584; ABclonal) were employed. Lung tissue sections (5 μm) were mounted on positively charged slides, deparaffinized, rehydrated through graded alcohols, and subjected to antigen retrieval. Following washes and blocking with BSA and hydrogen peroxide, the sections were incubated overnight at 4 °C with primary antibodies at dilutions of 1:300 for TGF-β1 and 1:100 for Vimentin. Subsequently, the sections were treated with an HRP-conjugated goat anti-mouse secondary antibody (Abcam, Cambridge, UK) for 2 h, and immunoreactivity was visualized using a DAB substrate kit (Thermo Scientific). Slides were counterstained with Mayer’s hematoxylin, dehydrated through graded alcohols, cleared in xylene, and mounted with DPX. The percentage area of positive staining was quantified using ImageJ software (version 1.53; National Institutes of Health, Bethesda, MD, USA). Negative controls were prepared by omitting the primary antibody incubation.

### Biochemical assays

Levels of malondialdehyde (MDA) and glutathione (GSH) in lung tissue homogenates were measured spectrophotometrically using commercial kits from Biodiagnostic (Giza, Egypt), following the manufacturer’s protocols.

Also, lung tissue levels of TNF-α, IL-1β, and c-AMP were measured using ELISA kits from CUSABIO (CSB-E11987r, Houston, USA) and MyBioSource (MBS825017 and MBS2700004, San Diego, USA), respectively. Absorbance was measured using the BioTek ELISA Reader (Winooski, USA). All were expressed as pg/mg protein.

### Western blot analysis

SIRT1 protein levels in lung tissue were evaluated using western blot analysis. Lung samples were homogenized, and the resulting lysates were centrifuged at 18,000 × g for 1 h at 4 °C to obtain the supernatants. Total protein was isolated following the manufacturer’s protocol, and its concentration was measured using the Coomassie (Bradford) assay reagent (Pierce, Rockford, IL, USA). Equal protein amounts were loaded onto 8% SDS-PAGE gels, separated using electrophoresis, and transferred onto nitrocellulose membranes. The membranes were blocked with 5% non-fat milk for 1 h and 30 min, then incubated overnight at 4 °C with the primary SIRT1 antibody (MA5-27217, Invitrogen, Waltham, USA) at a dilution of 1:1000. After washing with TBST, the membranes were incubated for 1 h at room temperature with the appropriate horseradish peroxidase (HRP)-conjugated secondary antibody at a dilution of 1:5000. Protein bands were visualized using the Odyssey Infrared Imaging System (LI-COR, USA). Band densities were quantified using ImageJ software (version 1.53; National Institutes of Health, Bethesda, MD, USA).

### Quantitative real-time polymerase chain reaction (qRT-PCR)

Quantitative real-time PCR (qRT-PCR) was employed to evaluate Rapgef3 gene expression in lung tissue. Total RNA was isolated using TRIzol reagent (Thermo Fisher Scientific, Waltham, MA, USA), and both RNA yield and purity were determined spectrophotometrically at 260 nm and by assessing the 260/280 nm ratio with a NanoDrop spectrophotometer (Thermo Fisher Scientific, TX, USA). Only samples with a purity ratio of ≥ 1.8 were included in the analysis. Complementary DNA (cDNA) was generated from 1 µg of RNA using the Qiagen RT-PCR kit (Qiagen, USA). qRT-PCR was conducted using Maxima SYBR Green/Fluorescein qPCR Master Mix (Thermo Scientific, USA) on a Rotor-Gene Q cycler (Qiagen, USA). Each 25 µl reaction mixture consisted of 12.5 µl SYBR Green Master Mix, 2 µl of forward and reverse primers, 5.5 µl RNase/DNase-free water, and 3 µl cDNA. The mixture was gently vortexed before amplification. The thermal cycling protocol included an initial activation at 95 °C for 10 min, followed by 45 cycles of denaturation at 95 °C for 10 s, annealing at 60 °C for 15 s, and extension at 72 °C for 15 s. Primer specificity was confirmed using melt-curve analysis. Gene-specific primers (Table 2) were designed via the PrimerQuest Tool based on sequences from PubMed (Entrez Gene) and synthesized by Invitrogen (CA, USA). Expression of Rapgef3 was normalized to β-actin, and relative expression was calculated using the 2 ^− ΔΔCT^ method.

**Table 2:** Primer sequences, accession numbers, and product sizes of the analyzed genes used for quantitative RT-PCR in lung tissue.

Gene of interest		Primer sequence	Reference sequence	Product size
β-actin	F	5′-AAGATCCTGACCGAGCGTGG-3′	NM_031144.3	327
	R	5′-CAGCACTGTGTTGGCATAGAGG-3′		
EPAC1 (gene name Rapgef3)	F	5′- GAGGCTCGGAGTCCTTAGTC − 3′	NM_001437876.1	158
	R	5′- GAGTCCCTAGCGCAGGAAAG-3′		

###  In silico molecular docking (MD)

The interaction of Cilostazol with transforming growth factor-β receptor I (TGF-β RI) and SIRT1 was predicted using AutoDock Vina and AutoDock Tools, following previously described protocols^63^. First, the Protein Data Bank (https://www.rcsb.org/pdb/home/home.do) was used to download the TGF beta RI and SIRT1 crystal structure (PDB code: 6B8Y and 4ZZJ, respectively). All water molecules and co-crystallized complexes were removed from the protein structures, and hydrogen atoms were added to adjust amino-acid ionization and tautomeric states. The three-dimensional structure of Cilostazol was retrieved from the ZINC database and chemically optimized by correcting bond orders, adding charges, incorporating hydrogens, and performing energy minimization. Both protein and ligand files were then converted into PDBQT format for use with the AutoDock software.

Molecular docking was carried out using AutoDock Vina at the binding sites corresponding to the D0A and 4TQ co-crystallized ligands. For TGF-β RI, the grid box was centered at X = 5.316784, Y = 8.883189, and Z = 5.092757. For SIRT1, the grid center coordinates were X = − 0.423893, Y = 44.524196, and Z = − 0.038714. The resulting docking poses and ligand–protein interactions within the binding pockets were analyzed and visualized using Chimera 1.15 and Discovery Studio Visualizer version 21.1.0.20298.

### Statistical analysis

Prior to performing statistical analyses, the normality of data distribution was assessed using the Shapiro-Wilk test, and the homogeneity of variances among groups was evaluated using Levene’s test. All datasets satisfied the assumptions of normality and equal variances; therefore, parametric analysis was conducted using one-way ANOVA followed by Tukey’s post hoc test for multiple comparisons between groups. Data processing and graphical visualization were carried out using GraphPad Prism version 6.01 (GraphPad Software, San Diego, CA, USA). Results are expressed as the mean ± standard error (SEM), and differences were considered statistically significant at *p* < 0.05.

##  Results

### Effect of Cilostazol on total and differential cell counts in BALF

Table 3 shows the effect of Cilostazol on AMIO-induced changes in total and differential cell counts in rats BALF. AMIO administration led to a significant increase (*P* < 0.001) in total cell counts in the BALF compared to the control group, indicating the development of acute inflammatory responses. In rats treated with cilostazol, total cell counts were significantly reduced (*P* < 0.001) relative to the AMIO group. Differential cell analysis revealed that neutrophils and lymphocytes were markedly elevated (*P* < 0.001) in AMIO-exposed rats, and this increase was significantly suppressed (*P* < 0.001) by cilostazol treatment.

**Table 3:** Effect of Cilostazol on AMIO-induced changes in total and differential cell counts in rats BALF.

Groups	Total cell count (cell per mm^3^) × 10^2^	Neutrophils (cell per mm^3^) × 10^2^	Lymphocytes (cell per mm^3^) × 10^2^
Normal	1.635 ± 0.027	0.6 ± 0.01	0.2 ± 0.006
AMIO	2.420 ± 0.004***	0.95 ± 0.017***	0.6 ± 0.009***
Cilostazol	1.91 ± 0.033^###^	0.78 ± 0.006^###^	0.3 ± 0.04^###^

BALF: bronchoalveolar lavage fluid, AMIO: Amiodarone. Data are presented as mean ± SEM. (*n* = 6). ***P < 0.001, compared with Normal control group. ^###^ *P* < 0.001, compared with AMIO group.

### Cilostazol attenuated AMIO-induced pulmonary histopathological alteration and lung index elevation

**Fig. 2:**
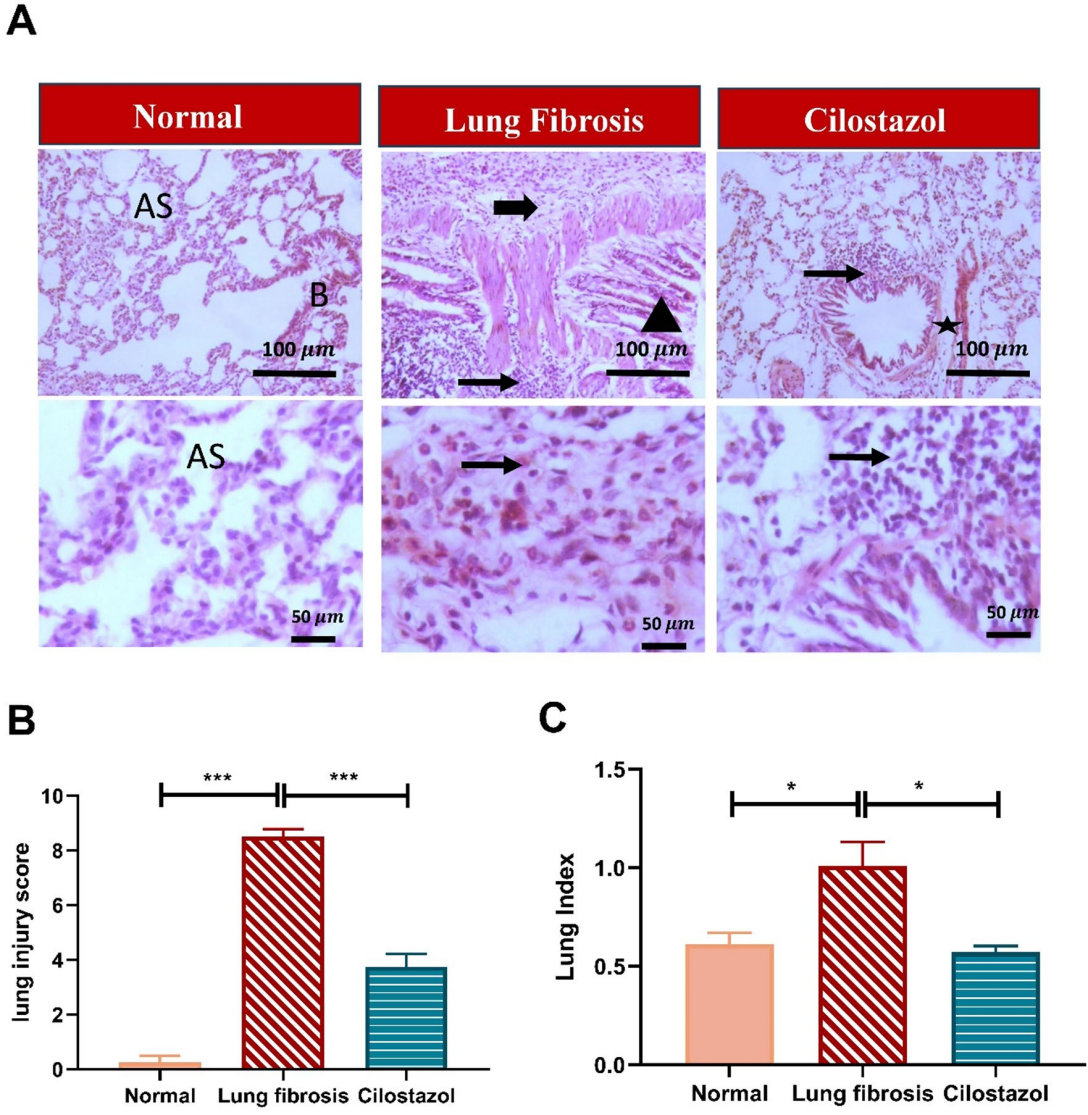
Cilostazol attenuated AMIO-induced pulmonary histopathological alteration and lung index elevation. (**A**) Photomicrographs of lung sections stained with hematoxylin and eosin (H&E). AS indicates intact alveolar sac, B indicates normal bronchiole, thick arrows indicate peribronchiolar fibrosis, thin arrows indicate inflammation, arrowheads indicate bronchiolar epithelial necrosis, and stars indicate perivascular edema. Image magnification 100x = Bar 100$\:\:{\upmu\:}\mathrm{m}$, 400x = Bar 50 $\:{\upmu\:}\mathrm{m}$. (**B**) Lung injury score. (**C**) Lung index. Data are presented as mean ± SEM. (*n* = 6). *P < 0.05 and ***P < 0.001.

As illustrated in Fig. 2, control group showed normal histological appearance of alveolar sac with intact bronchiolar wall. AMIO group showed extensive peribronchiolar fibrosis associated with marked cellular infiltrates composed of abundant lymphocytes, plasma cells, macrophages and mild to moderate numbers of neutrophils and eosinophils extending and replacing the pulmonary sections and compressing the adjacent alveoli. Cilostazol treatment demonstrated focal moderate perivascular and peribronchiolar cellular infiltrates, predominantly composed of lymphoplasmacytic cells with mild fibrosis and perivascular edema, along with a marked improvement in lung architecture, as evidenced by a significant attenuation in the lung injury compared with the AMIO group (*P* ≤ 0.001). The rats from the AMIO group showed a notable elevation in the lung/body weight index by 64.7% (*P* < 0.05) as compared to those from the normal control group. Cilostazol treatment caused a substantial reduction in lung/body weight index by approximately 43.4% (*P* < 0.05) as compared to AMIO group. Moreover, no significant difference was observed between the Cilostazol group and the normal control group.

###  Cilostazol mitigates AMIO-induced pulmonary oxidative stress and inflammation

**Fig. 3:**
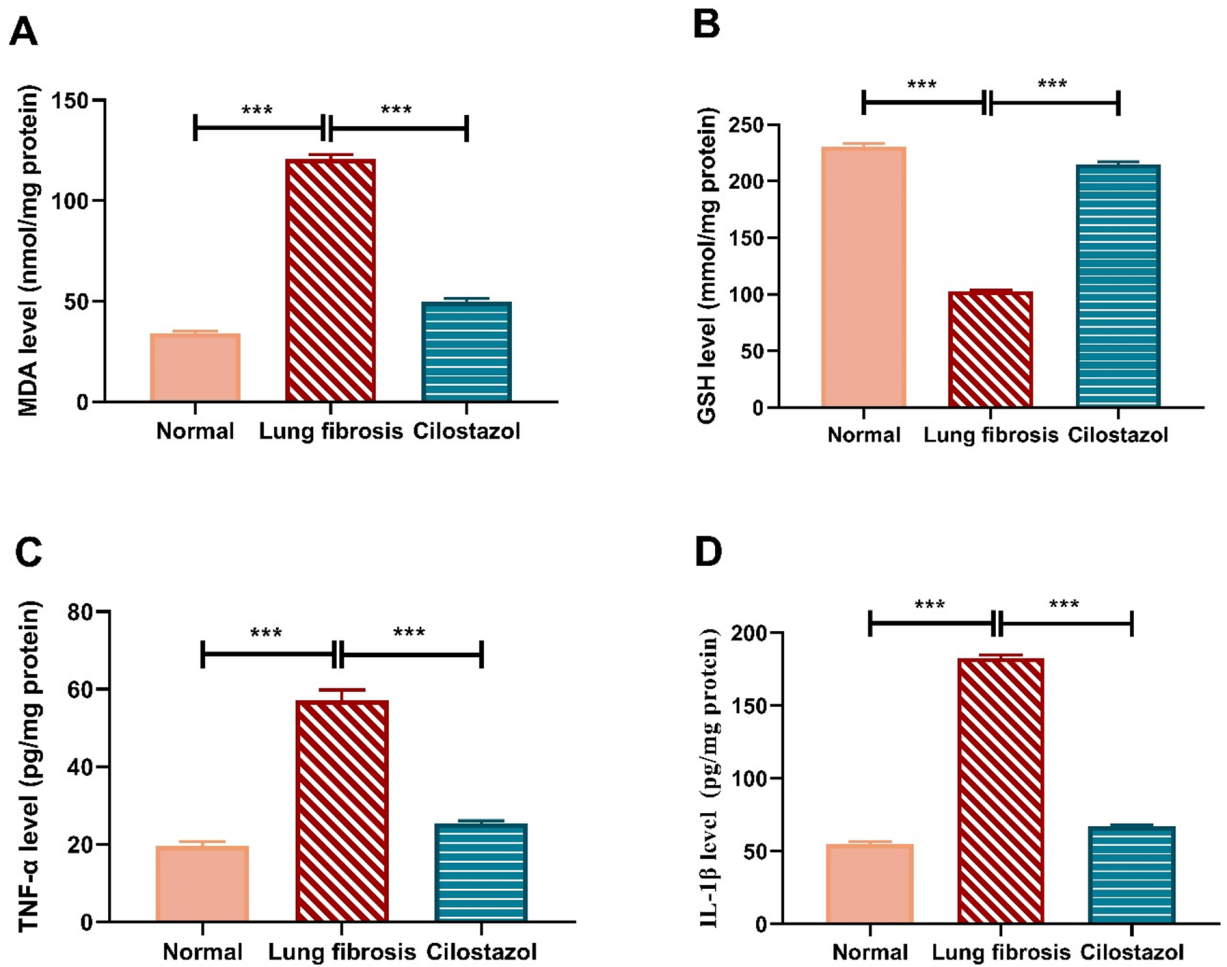
Cilostazol mitigates AMIO-induced pulmonary oxidative stress and inflammation. The effect of Cilostazol on lung MDA content (**A**), lung GSH content (**B**), lung TNF-α level (**C**), and lung IL-1β level (**D**). Data are presented as mean ± SEM. (*n* = 6). ***P < 0.001.

As shown in (Fig. 3A and B), Administration of AMIO led to a pronounced increase in lung MDA levels, approximately 3.5-fold, accompanied by a substantial decrease in lung GSH content of nearly 2.2-fold relative to the normal control group. In contrast, co-treatment with AMIO and Cilostazol resulted in a significant reduction of lung MDA levels by about 2.4-fold and a notable restoration of lung GSH content, showing an approximate 2-fold increase compared to the AMIO-only group.

As shown in (Fig. 3C and D), AMIO administration resulted in a marked increase in pulmonary TNF-α and IL-1β levels, approximately 2.8-fold and 3.3-fold, respectively, compared to the normal control group. Conversely, co-administration of AMIO with Cilostazol significantly reduced TNF-α and IL-1β levels in the lung by approximately 2.2-fold and 2.7-fold, respectively, relative to the AMIO-only group.

### Cilostazol attenuates AMIO-induced reduction in pulmonary cAMP, and EPAC1

**Fig. 4:**
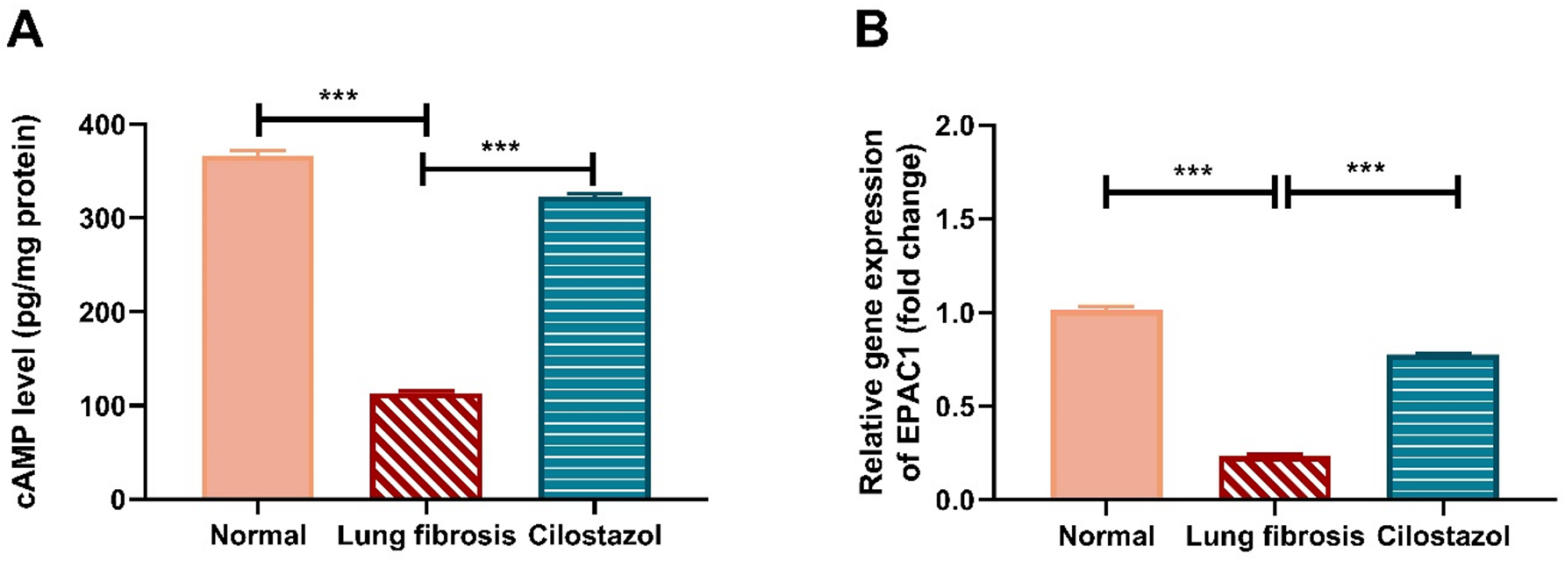
Cilostazol attenuates AMIO-induced reduction in pulmonary cAMP, and EPAC1. (**A**) cAMP level (pg/mg protein measured by ELISA. (**B**) Relative gene expression of EPAC1 measured by RT-PCR. Data are presented as mean ± SEM. (*n* = 6). ***P < 0.001.

As shown in (Fig. 4), Administration of AMIO caused a significant reduction in pulmonary cAMP, and EPAC1 levels, by approximately 3.2, and 4.3-fold, respectively, relative to the normal control group. In contrast, co-treatment with AMIO and Cilostazol markedly increased lung cAMP, and EPAC1 levels by about 2.9, and 3.3-fold, respectively, compared to the AMIO-only group.

###  Cilostazol activates SIRT1 in AMIO-induced pulmonary fibrosis model

**Fig. 5:**
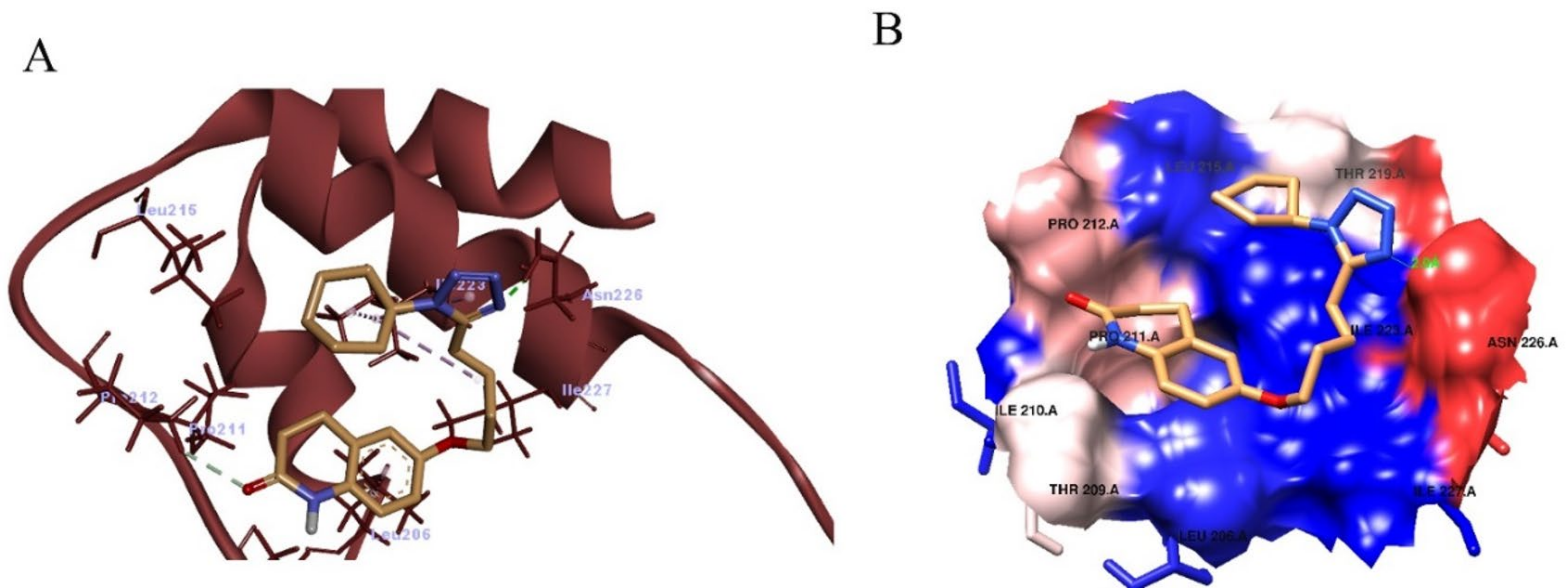
Molecular docking analysis of cilostazol with SIRT1. (**A**) Participating amino acids in the interaction of SIRT1 (PDB code: 4ZZJ) with cilostazol using Discovery Studio Visualizer. (**B**) Hydrophobic interactions between cilostazol and SIRT1 pocket using Chimera.

The impact of cilostazol on SIRT1 protein was assessed using AutoDock Vina version 1.1.2^64^. The binding affinity was − 5.1 Kcal/mol when cilostazol interacted with SIRT1 in the site of 4TQ. Cilostazol was able to form hydrogen bond with ASN226. Also, the binding of cilostazol with SIRT1 was promoted by hydrophobic interactions with LEU206, ILE227, ILE223, LEU215, and ILE210. The results of docking were illustrated in Fig. 5 and Table 4.

**Table 4:** The interacting polar residues, hydrophobic interactions and binding affinities of Cilostazol with SIRT1.

Molecular target and PDB code	Hydrogen bond analysis		Amino acids involved in lipophilic analysis*	2D ligand/protein interaction	Binding affinity (kcal/mol)
	Amino acid involved in H bond	Distance (Å)			
SIRT1 (AZZJ)	ASN226	2	LEU206, ILE227, ILE223, LEU215, and ILE210		− 5.1

**Fig. 6:**
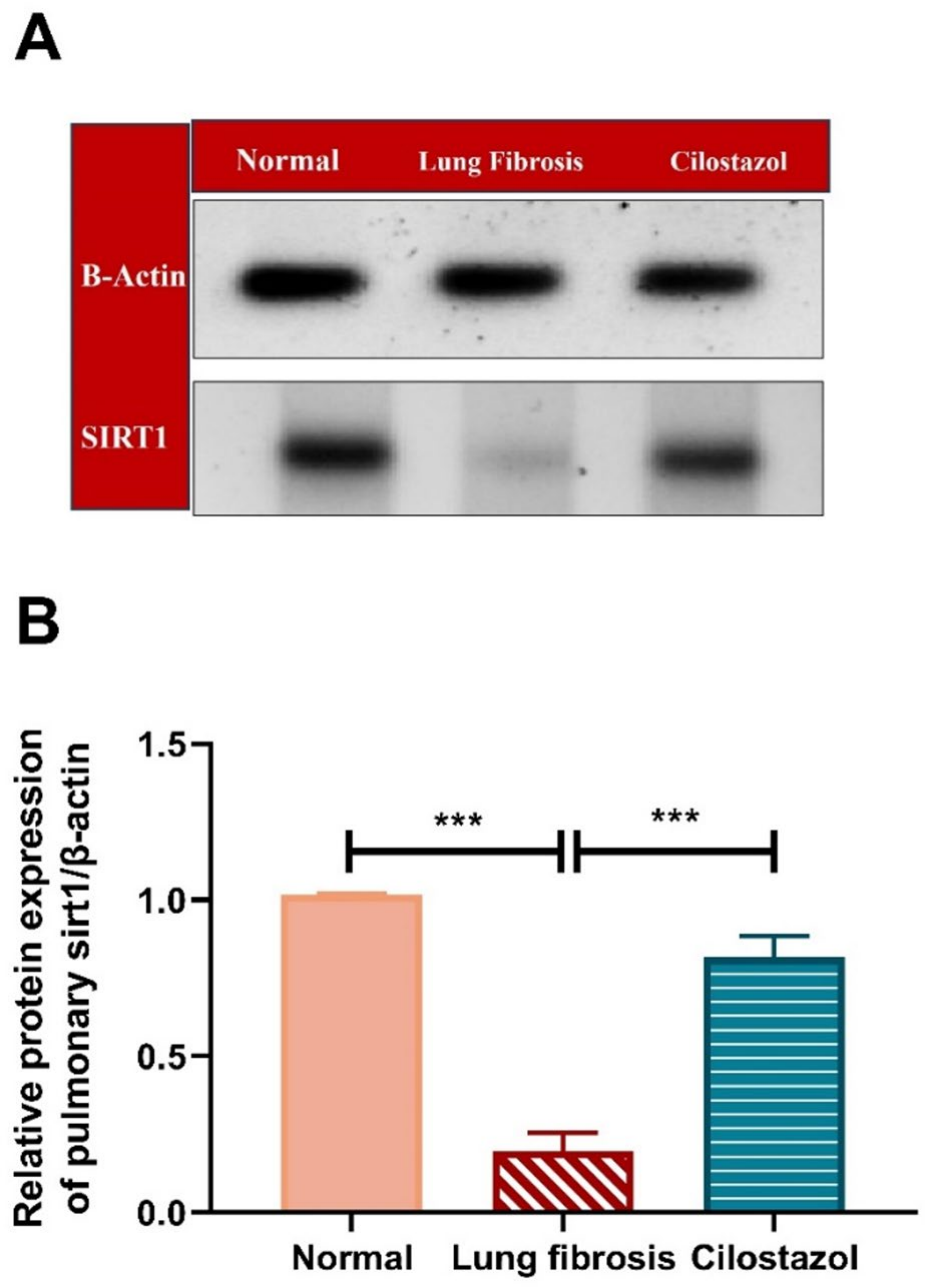
Cilostazol activates SIRT1 in AMIO-induced pulmonary fibrosis model. (**A**) effect of cilostazol on pulmonary level of SIRT1 protein expression was assessed with western blotting technique. (**B**) Quantitative analysis of western blot. Data are presented as mean ± SEM. (*n* = 6). ***P < 0.001.

As shown in (Fig. 6), Administration of AMIO caused a significant reduction in pulmonary SIRT1 level, by approximately 5.2-fold, relative to the normal control group. In contrast, co-treatment with AMIO and Cilostazol markedly increased lung SIRT1 level by about 4.2-fold, compared to the AMIO-only group.

### Cilostazol attenuated AMIO-induced pulmonary fibrosis

**Fig. 7:**
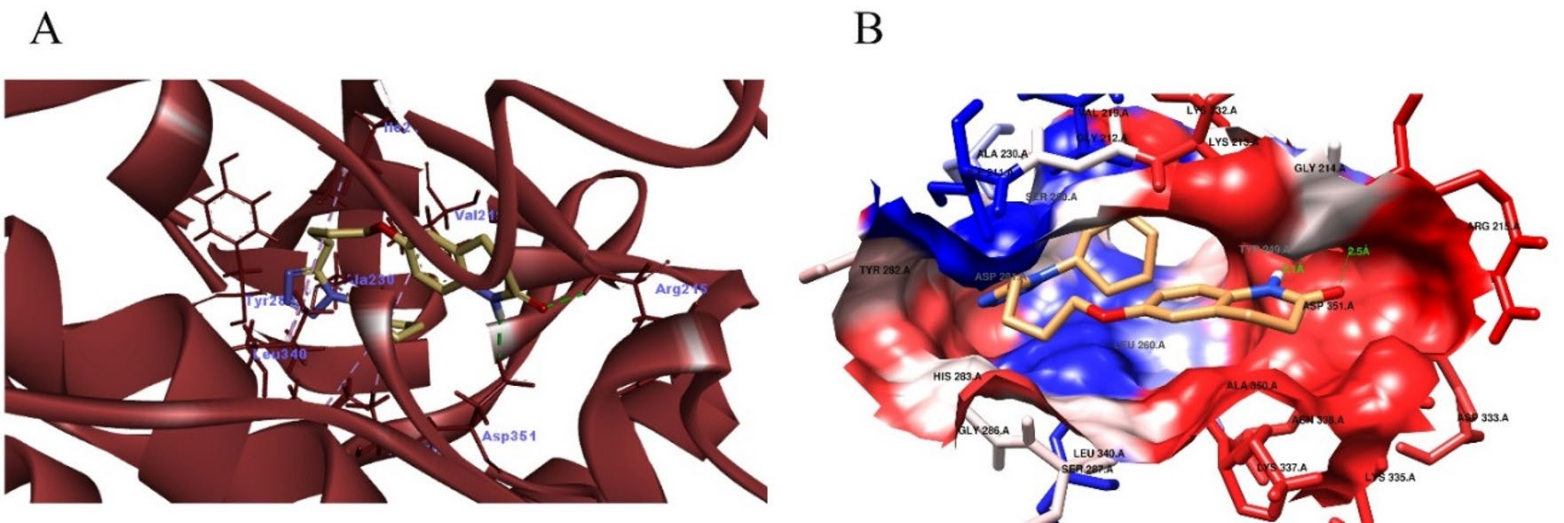
Molecular docking analysis of cilostazol with TGF-β RI. (**A**) Participating amino acids in the interaction of TGF beta RI (PDB code: 6B8Y) with cilostazol using Discovery Studio Visualizer. (**B**) Hydrophobic interactions between cilostazol and TGF beta RI pocket using Chimera.

The impact of cilostazol on TGF-β RI protein was assessed using AutoDock Vina version 1.1.2^64^. The binding affinity was − 8.9 Kcal/mol when cilostazol interacted with TGF beta RI in the site of D0A. Cilostazol was able to form hydrogen bond with ARG215 and ASP351. Also, the binding of cilostazol with TGF beta RI was promoted by hydrophobic interactions with LEU340, LEU260, ALA230, VAL219, and GLY286. Figure 7 and Table 5.

**Table 5:** The interacting polar residues, hydrophobic interactions and binding affinity of cilostazol with TGF beta RI.

Molecular target and PDB code	Hydrogen bond analysis		Amino acids involved in lipophilic analysis*	2D ligand/protein interaction	Binding affinity (kcal/mol)
	Amino acid involved in H bond	Distance (Å)			
TGF beta RI (6B8Y)	ARG215	2.5	LEU340, LEU260, ALA230, VAL219, and GLY286		−8.9
	ASP351	2.1			

**Fig. 8:**
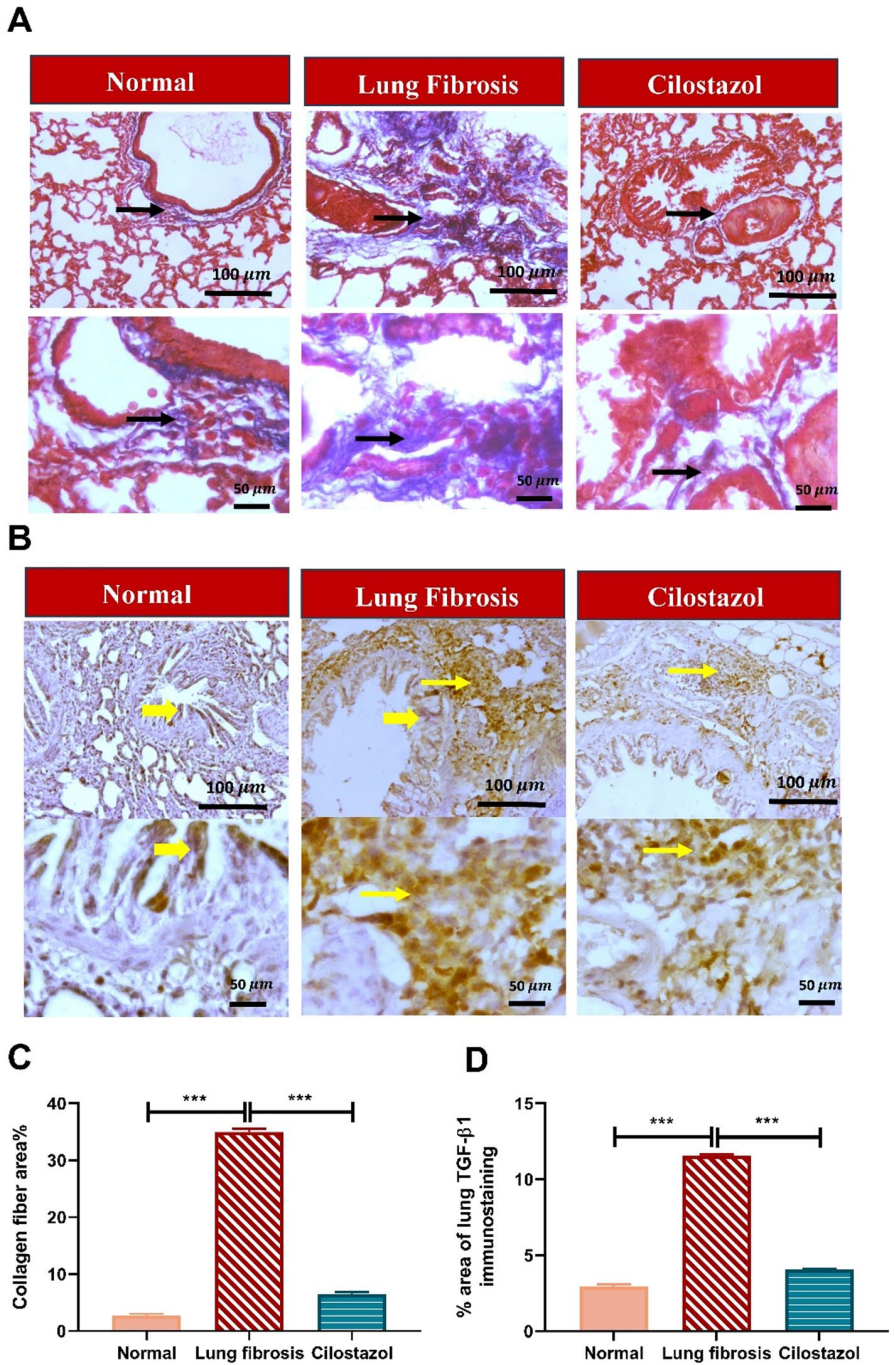
Cilostazol attenuated AMIO-induced pulmonary fibrosis. (**A**) The photomicrographs of lung sections stained with Masson’s trichrome. Thin black arrows indicate bluish stained collagen deposition. (**B**) The photomicrographs of immunostained-lung sections against TGF-β1. Thin yellow arrows indicate positive inflammatory cells, and thick yellow arrows indicate positive bronchiolar cells. (**C**) Collagen fiber area %. (**D**) % area of lung TGF-β1 immunostaining. Image magnification 100x = Bar 100$\:\:{\upmu\:}\mathrm{m}$, 400x = Bar 50 $\:{\upmu\:}\mathrm{m}$. Data are presented as mean ± SEM. (*n* = 6). ***P < 0.001.

Images of the lung tissue stained with Masson’s trichrome showed normal deposition of collagen around blood vessels of pulmonary sections in normal rats. The administration of AMIO caused a remarkable increase in bluish-stained alveolar peribronchiolar collagen deposition by 293% as compared with the normal control group. In contrast, treatment with cilostazol resulted in mild perivascular and peribronchiolar deposition of fine collagen fiber strands, accompanied by a 64.96% reduction in fibrosis compared with the AMIO group (Fig. 8A and C).

Furthermore, the effect of Cilostazol on the expression of the fibrotic marker TGF-β1 was further assessed by immunohistochemistry technique^65^. Immunohistochemical analysis of lung sections demonstrated minimal TGF-β1 immunopositivity in bronchiolar and alveolar cells of the normal group. In contrast, the AMIO-treated group showed a pronounced increase in the TGF-β1–positive area, approximately 3.9-fold higher than the normal control. Notably, daily oral administration of Cilostazol resulted in a marked reduction of TGF-β1 immunopositivity in the lung, with an approximate 2.8-fold decrease compared to the AMIO-only group (Fig. 8B and D).

### Cilostazol attenuates AMIO-induced epithelial–mesenchymal transition

**Fig. 9:**
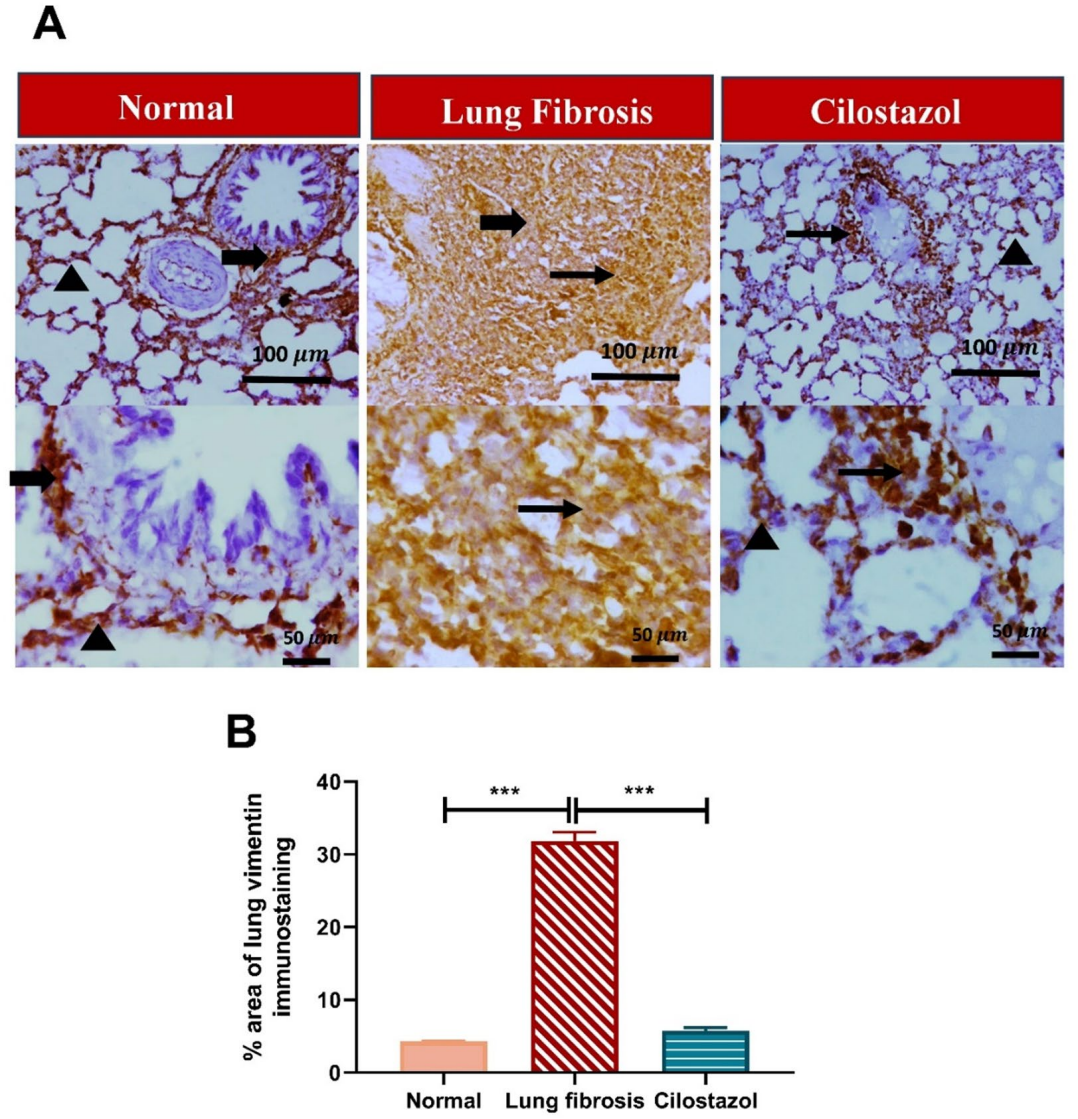
Cilostazol attenuates AMIO-induced epithelial–mesenchymal transition. (**A**) The photomicrographs of immunostained-lung sections against Vimentin. (**B**) % area of lung Vimentin immunostaining. Thin arrows indicate positive inflammatory cells, thick arrows indicate positive fibrous tissue cells, and arrowheads indicate positive alveolar cells. Image magnification 100x = Bar 100$\:\:{\upmu\:}\mathrm{m}$, 400x = Bar 50 $\:{\upmu\:}\mathrm{m}$. Data are presented as mean ± SEM. (*n* = 6). ***P < 0.001.

Analysis of Vimentin-immunostained lung sections from the AMIO group revealed a pronounced increase in the positively stained area, approximately 7.5-fold higher than that observed in the normal control group. Remarkably, daily oral administration of Cilostazol led to a significant reduction in Vimentin immunopositivity, showing an approximate 5.5-fold decrease compared to the AMIO-only group (Fig. 9).

###  Multivariable data analyses

**Fig. 10:**
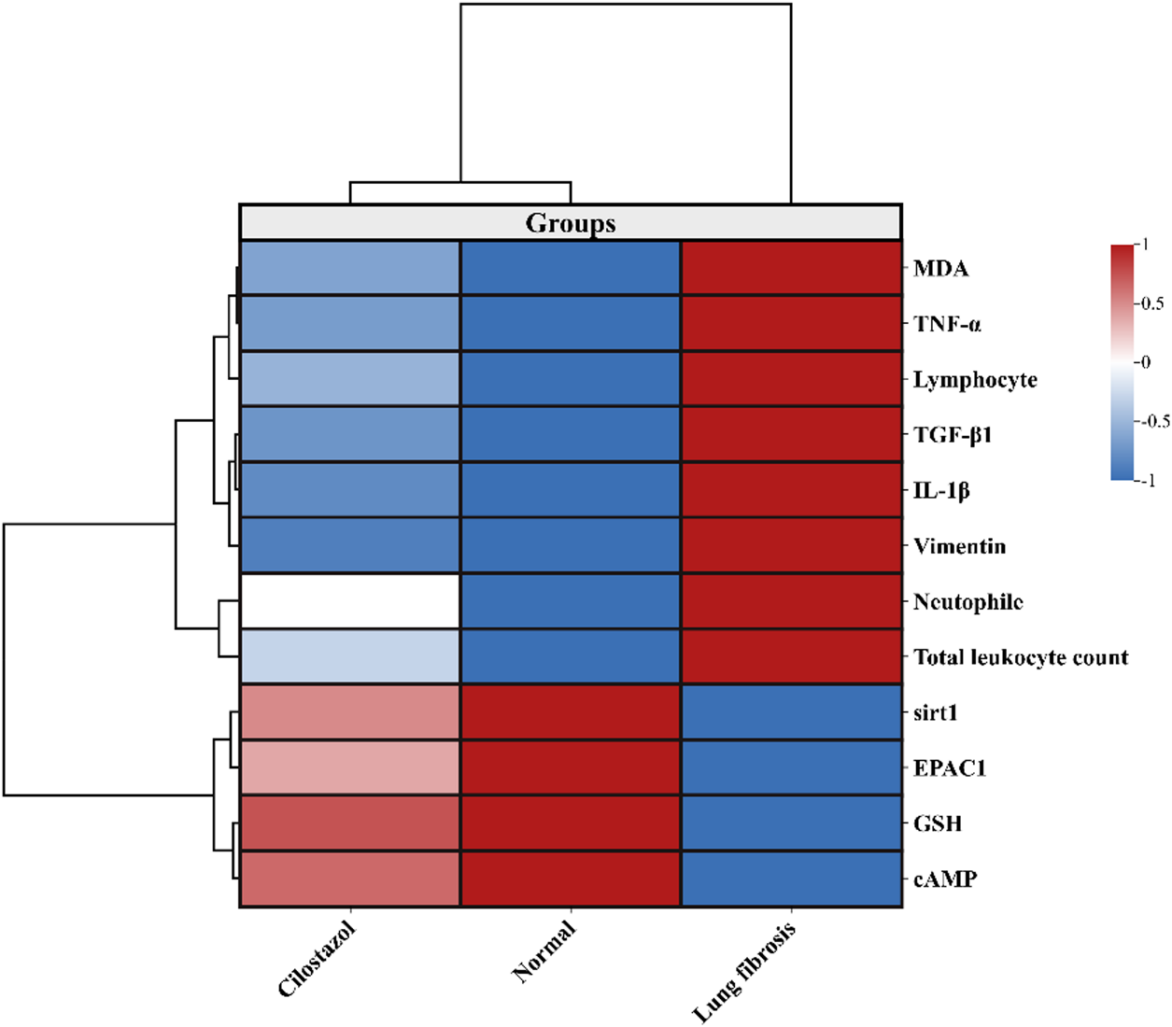
Heatmap representing hierarchical clustering of biochemical, oxidative stress, and molecular markers across normal, lung fibrosis, and cilostazol groups. Color gradients indicate relative expression levels (red = upregulation, blue = downregulation; Z-score normalized). The lung fibrosis group exhibited elevated levels of markers (MDA, TNF-α, lymphocytes, TGF-β1, IL-1β, Vimentin, neutrophiles, and total leukocyte count). In contrast, these markers were markedly reduced in normal and cilostazol groups. Conversely, GSH, Sirt1, EPAC1 and cAMP exhibited upregulation in the cilostazol group., reflecting improved redox balance, migitated inflammation and fibrosis with treatment.

Hierarchical clustering and heatmap analysis of the biochemical and molecular parameters revealed clear distinctions among the experimental groups (Normal, lung fibrosis, and cilostazol-treated). The dark red shading indicated that the lung fibrosis group had elevated levels of markers, including MDA, TNF-α, lymphocytes, TGF-β1, IL-1β, Vimentin, neutrophils, and total leukocyte count. In contrast, the control group showed a marked decrease in these markers (blue gradient), suggesting that cilostazol treatment attenuated lung fibrosis, reduced inflammation, and mitigated oxidative stress. Meanwhile, GSH, SIRT1, EPAC1, and cAMP were upregulated in the normal group. The clustering patterns visually demonstrate the extent to which cilostazol modulates inflammatory and fibrosis markers as well as oxidative stress (Fig. 10).

## Discussion

Lung fibrosis is a serious side effect of the antiarrhythmic drug AMIO, characterized by the excessive deposition of extracellular matrix (ECM) proteins, which leads to impaired lung function^66^. Cilostazol, a phosphodiesterase III inhibitor, has shown promising effect in attenuating fibrotic process through its modulation of cAMP signaling cascade and its downstream pathways^67,68^. This study investigates the antifibrotic potential of cilostazol, focusing on its ability to elevate cAMP levels, which subsequently activate both Epac/Rap1 and SIRT1/ROS, as well as its impact on the TGF-β–induced EMT.

The results demonstrated a marked infiltration of inflammatory cells, particularly neutrophils and lymphocytes, accompanied by deterioration of alveolar epithelial structures following AMIO administration, as indicated by the significant increase in total and differential cell counts in BALF. These observations are consistent with previous reports describing AMIO-induced pulmonary inflammation and tissue injury^69–71^. Treatment with cilostazol markedly alleviated these alterations, as evidenced by the significant reduction in total and differential cell counts, reflecting its strong anti-inflammatory potential. This finding is in line with earlier studies emphasizing the anti-inflammatory and endothelial-protective properties of cilostazol in various models^72,73^.

Consistent with the cellular findings, biochemical analysis revealed that AMIO administration caused a substantial upregulation of pro-inflammatory cytokines, as evidenced by the significant increases in TNF-α and IL-1β levels in lung tissue in a line with several studies^70,74^. In contrast, cilostazol treatment significantly attenuated this cytokine surge, markedly decreasing TNF-α and IL-1β expression compared with the AMIO group, consistent with previous findings^75,76^. These results highlight the potent cytokine-modulating and anti-inflammatory actions of cilostazol, which likely contribute to its protective role against AMIO-induced lung injury.

Histopathological examination further supported these findings, where AMIO exposure resulted in extensive peribronchiolar fibrosis and dense cellular infiltrates composed mainly of lymphocytes, macrophages, and neutrophils, leading to distortion of the pulmonary architecture. In contrast, cilostazol treatment markedly ameliorated these pathological alterations, restoring near-normal alveolar structure and reducing inflammatory infiltration and fibrosis.

Furthermore, AMIO-induced lung injury was associated with a significant elevation in the lung/body weight index, reflecting edema and fibrotic remodeling, consistent with previously reported models of drug-induced pulmonary fibrosis^69,77^. Cilostazol administration significantly reduced this index, suggesting attenuation of tissue injury and fibrotic progression. These findings were further supported by Masson’s trichrome staining, which demonstrated a profound increase in collagen deposition following AMIO exposure, while cilostazol markedly reduced collagen accumulation by approximately 65%, confirming its anti-fibrotic potential. Collectively, these findings demonstrate that cilostazol provides substantial protection against AMIO-induced pulmonary inflammation and fibrosis, likely through suppression of inflammatory cell recruitment, downregulation of pro-inflammatory cytokines, and inhibition of collagen deposition, thereby preserving lung architecture and function.

cAMP is a key second messenger that regulates multiple cellular processes, including fibrosis^78^. Cilostazol, by inhibiting phosphodiesterase III, increases intracellular cAMP levels, which has been shown to counteract the profibrotic effects of AMIO through inhibiting fibroblast proliferation and differentiation into myofibroblasts^79^. Indeed, the present study revealed a significant restoration of lung cAMP and this comes in accordance with previous studies reporting increased cAMP in various tissues^80,81^. Elevated cAMP levels suppress the expression of profibrotic markers such as vimentin, which are crucial for myofibroblast function and fibrosis development. Additionally, elevated cAMP upregulates E-cadherin, thereby supporting cell-cell adhesion and potentially preventing TGF-β-induced EMT, a critical step in fibrosis progression^78^. These effects are likely mediated through downstream activation of Epac/Rap1 and SIRT1/ROS signaling pathways, highlighting the multifaceted antifibrotic mechanisms of cilostazol^58,82^.

The effect of cilostazol on Epac1 expression was further evaluated, and our study demonstrated an upregulation of Epac1 in cilostazol-treated lung tissues, consistent with previous report^67^. Epac1 has been identified as a key mediator of the antifibrotic effects associated with elevated intracellular cAMP levels^34^. By increasing cAMP, cilostazol activates Epac1, which subsequently modulates Rap1 activity. Activation of Epac1/Rap1 signaling pathway effectively attenuates key profibrotic processes, including fibroblast proliferation, myofibroblast differentiation, and collagen deposition^82^. These antifibrotic effects of Epac1 are believed to be mediated primarily through suppression of TGF-β/SMAD signaling pathway^83^. TGF-β is a key cytokine involved in lung fibrosis, primarily through its role in promoting EMT, leading to downregulation the expression of epithelial markers such as E-cadherin and upregulation of mesenchymal markers like vimentin^84^. This transition enhances the proliferation, and differentiation of fibroblasts into myofibroblasts, which are responsible for excessive deposition of ECM proteins, thereby exacerbating pulmonary fibrosis^85^. Our study revealed that cilostazol counteracts the fibrotic effects of AMIO by reducing TGF-β expression, potentially offering therapeutic insights for managing PF.

SIRT1, a member of the sirtuin family, is a key regulator of cellular metabolism, oxidative stress response, and fibrosis-related signaling. Its activation plays a crucial role in preserving redox balance, suppressing pro-fibrotic gene expression, and maintaining cellular homeostasis^86^. The present study demonstrated that cilostazol enhances SIRT1 expression and activity, thereby modulatingROS generation and mitigating oxidative injury. Consistent with findings from hepatic fibrosis models, in which cilostazol-induced SIRT1 activation suppressed ROS production and downregulated inflammatory cytokines such as NF-κB and TNF-α, as well as fibrotic mediators such as TGF-β1^58,87^, the current results suggest that SIRT1 upregulation contributes significantly to the antioxidant, anti-inflammatory, and antifibrotic effects of cilostazol. Therefore, restoration of SIRT1 expression may represent a central mechanism underlying the protective action of cilostazol against AMIO-induced PF.

Oxidative stress also plays a crucial role in the pathogenesis of pulmonary fibrosis, driving disease progression through both cellular and molecular damage^88^. AMIO is known to induce pulmonary toxicity by significantly increasing ROS level. This surge in ROS is characterized by elevated MDA levels, a biomarker of lipid peroxidation, alongside depletion of GSH, an essential intracellular antioxidant^89^. The resulting imbalance in redox homeostasis triggers oxidative damage and inflammatory responses in lung tissues, contributing to the progression and exacerbation of PF^90^. Conversely, cilostazol treatment reduced MDA levels and enhanced GSH content, indicating its antioxidant potential in mitigating oxidative stress associated with PF. These findings are in agreement with previous studies highlighting cilostazol’s ability to attenuate both oxidative stress and inflammation^91–93^.

To place the present findings in context, it is important to compare them with those reported for established antifibrotic therapies. Pirfenidone is a well-known antifibrotic agent approved for the treatment of IPF^94^. Previous experimental and clinical studies have demonstrated that pirfenidone attenuates lung fibrosis through several mechanisms, including suppression of transforming growth factor-β (TGF-β) signaling, inhibition of fibroblast proliferation, and reduction of collagen deposition^95,96^. In the present study, cilostazol significantly ameliorated AMIO-induced lung fibrosis as evidenced by improvement in histopathological alterations and modulation of fibrotic and inflammatory markers. These findings are consistent with the antifibrotic effects reported for pirfenidone in experimental pulmonary fibrosis models. Although pirfenidone was not included as a reference drug in the current experimental design, the observed protective effects of cilostazol suggest that modulation of cAMP signaling and its downstream pathways may represent a promising therapeutic strategy for the attenuation of PF. Further studies comparing cilostazol directly with established antifibrotic agents such as pirfenidone are warranted to better clarify its relative therapeutic efficacy.

The present study provides preliminary evidence for the protective effects of Cilostazol against AMIO-induced PF and highlights its potential antifibrotic mechanisms; however, several limitations should be acknowledged. First, the study relied on a single animal model of lung fibrosis induced by AMIO in rats, which may limit the generalizability of the findings. In humans, PF can arise from diverse etiologies, including IPF, exposure to fibrogenic agents such as bleomycin, or other drug- and radiation-induced causes, and the underlying fibrotic mechanisms may vary across these conditions. Therefore, future studies using multiple experimental models are needed to confirm the broader applicability of cilostazol. Second, functional assessments of lung physiology, such as blood gas analysis, airway opening pressure, and lung compliance, were not performed; inclusion of these measurements would provide additional insight into the physiological relevance of the observed protective effects. Another limitation is the absence of a standard antifibrotic drug as a positive control group, such as pirfenidone, which would have enabled direct comparison of therapeutic efficacy. Finally, further mechanistic investigations employing approaches such as molecular knockdown or overexpression strategies, detailed signaling pathway analyses, and single-cell transcriptomic profiling are warranted to better elucidate the molecular networks through which cilostazol mitigates PF. In addition, clinical studies will be necessary to evaluate the translational potential, safety, and therapeutic efficacy of cilostazol in patients with PF.

**Fig. 11:**
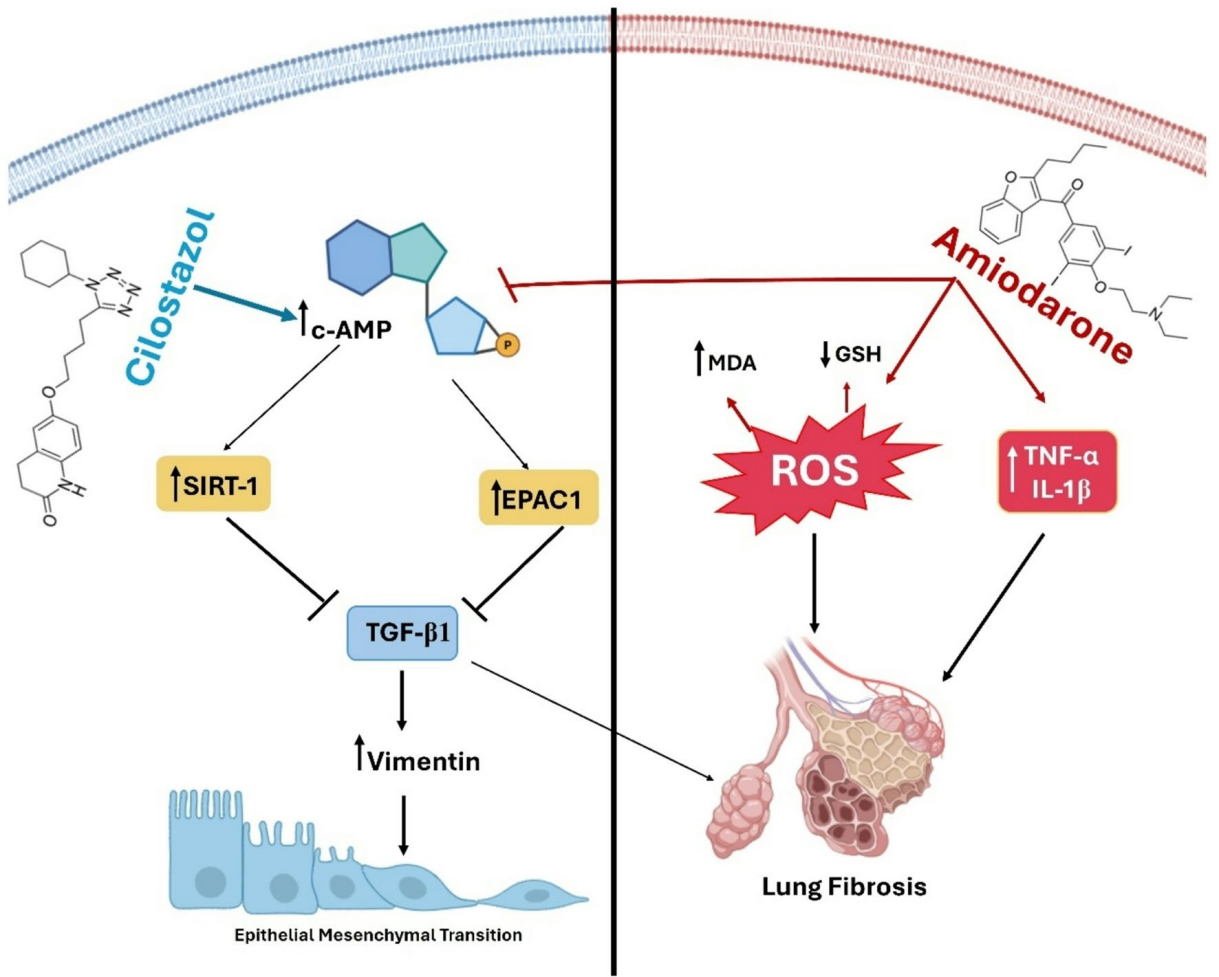
Proposed mechanism of Cilostazol action. Cilostazol attenuates amiodarone induced lung fibrosis by modulating cAMP/TGF-β1 pathway, which in turn inhibits transcription or translation of other downstream genes that are normally essential for inflammation, oxidative stress, fibrosis and epithelial-mesenchymal transition.

Collectively, these findings demonstrate that cilostazol provides substantial protection against AMIO-induced pulmonary inflammation and fibrosis, likely through suppression of inflammatory cell recruitment, downregulation of pro-inflammatory cytokines, elevation of cAMP, activation of Epac1/Rap1 and SIRT1/ROS pathways, inhibition of TGF-β–induced EMT, mitigation of oxidative stress, and reduction of collagen deposition, thereby preserving normal lung architecture and function (Fig. 11).

## Supplementary Information

Below is the link to the electronic supplementary material.

Supplementary Material 1

Supplementary Material 2

## Data Availability

The datasets used and/or analyzed during the current study are available from the corresponding author on reasonable request.
