# Supplementary figures and images for: Cilostazol mitigates amiodarone-induced pulmonary toxicity and fibrosis by regulating the cAMP/TGF-β1 pathway-mediated epithelial-to-mesenchymal transition in rats
Source: Sci Rep. 2026 May 13;16:15055. doi: 10.1038/s41598-026-45341-3 (PMC13171918; doi:10.1038/s41598-026-45341-3)

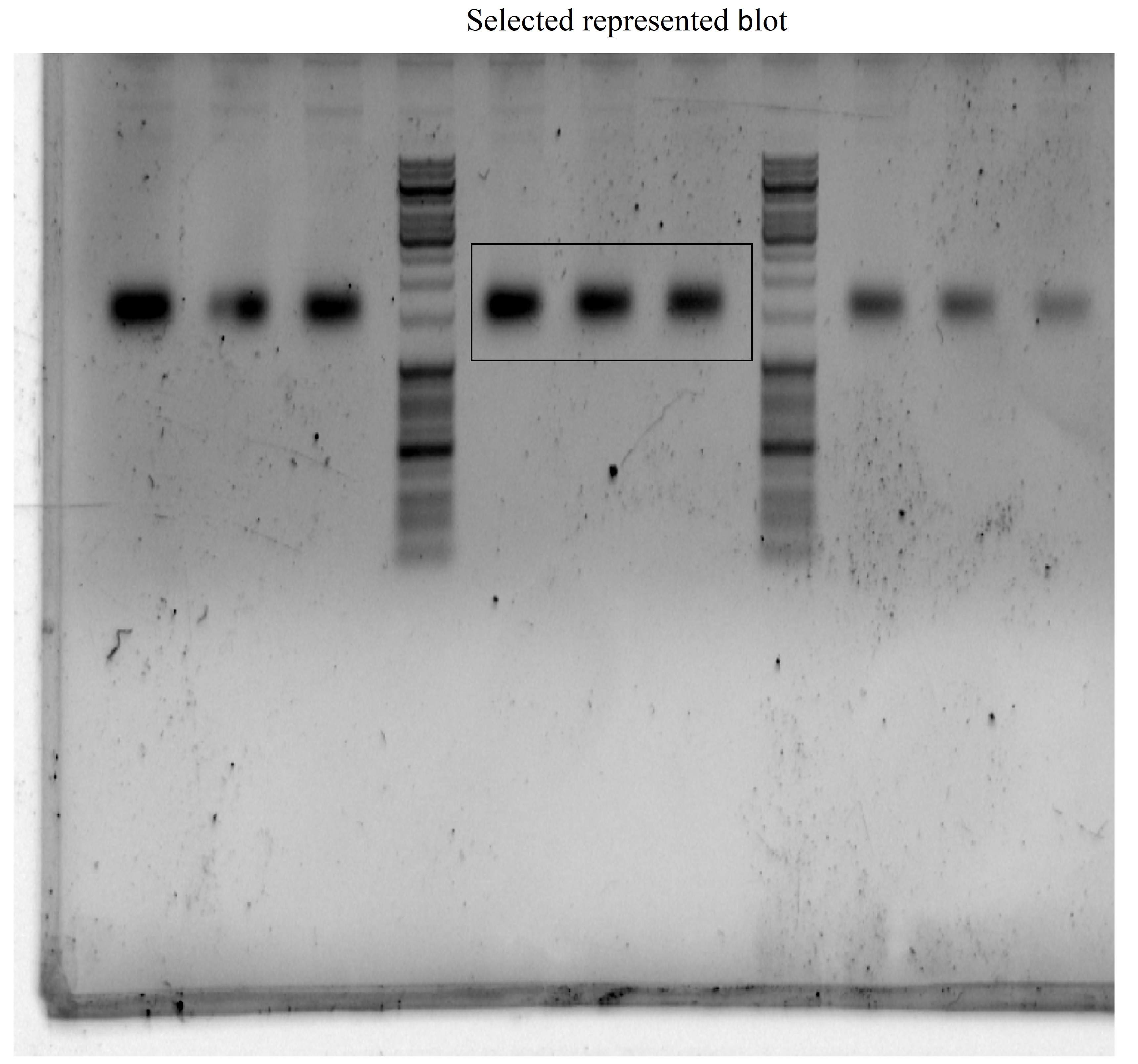

Supplement: Supplementary file 1 — Supplementary Material 1 [file 41598_2026_45341_MOESM1_ESM.jpg]

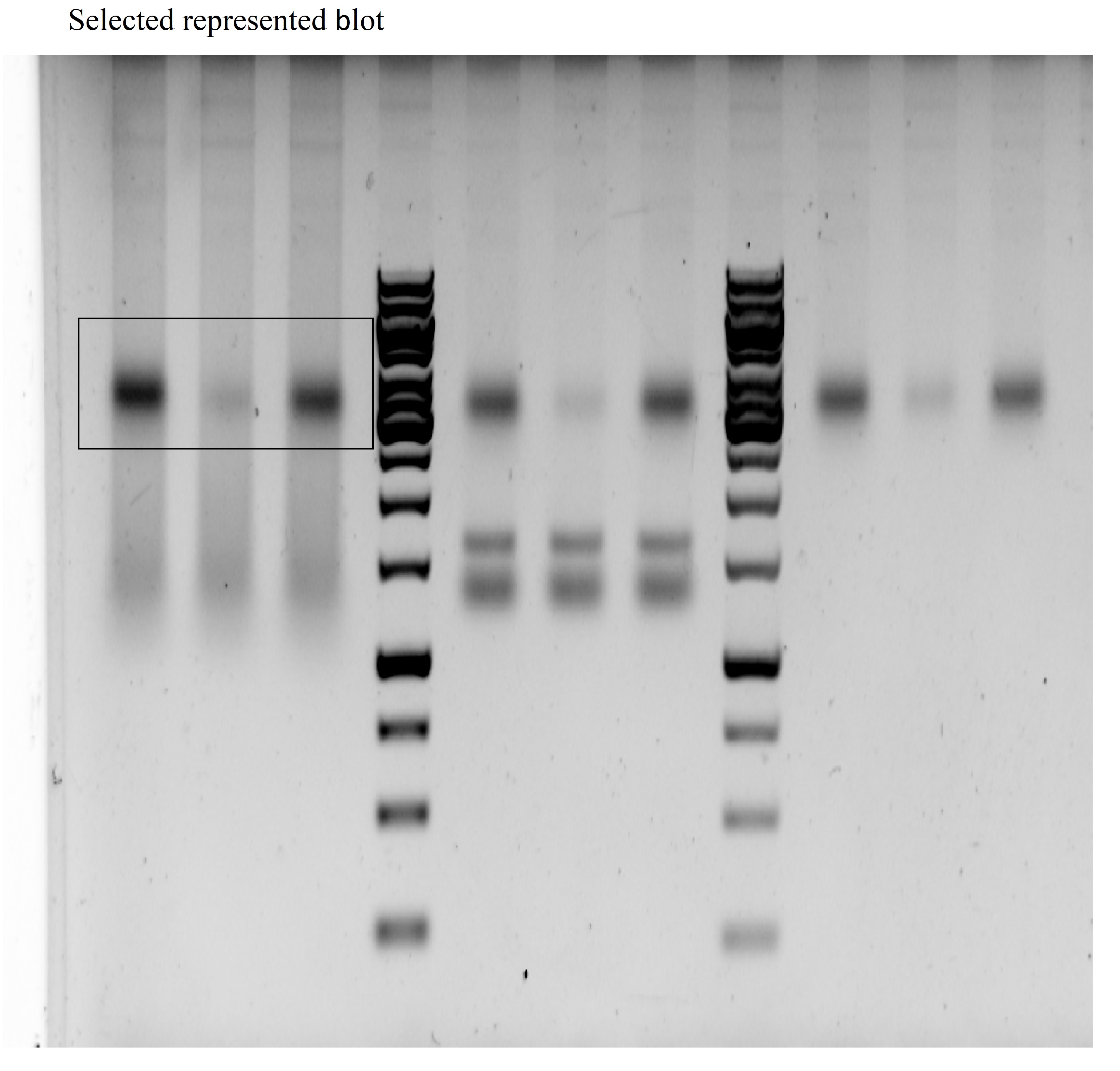

Supplement: Supplementary file 2 — Supplementary Material 2 [file 41598_2026_45341_MOESM2_ESM.jpg]
